# A review of the CTRC-AACR San Antonio Breast Cancer Symposium 2012

**DOI:** 10.3332/ecancer.2013.291

**Published:** 2013-02-11

**Authors:** Giuseppe Curigliano

**Affiliations:** Division of Medical Oncology, Department of Medicine, European Institute of Oncology, Via Ripamonti 435, 20141 Milan, Italy

## Abstract

The annual San Antonio Breast Cancer Symposium (SABC) is a multidisciplinary conference that covers basic molecular and cellular biology, epidemiology, diagnosis and treatment of all types of breast cancer and premalignant breast disease. In 2012, this meeting was held at the Henry B. Gonzalez Convention Center in San Antonio, Texas, United States, from 4 to 8 December.

The symposium consisted of lectures within six general sections covering a range of topics in breast cancer research. These included discussions on research of breast cancer posters, a large number of specialist sessions, and several minisymposia. The report below describes much of the research presented in those general lecture sessions.

The abstracts of all papers and posters presented at this conference have been published in *Cancer Research* as a supplement: *Cancer Research* (2012) **72(24)**, 1s–608s.

## Wednesday, 5 December GENERAL SESSION 1

### Letrozole versus tamoxifen for lobular carcinoma: Results from the BIG 1–98 trial

Over the last decade, the Breast International Group (BIG) 1–98 trial has generated significant quantities of data on the effectiveness of the aromatase inhibitor, letrozole, compared with tamoxifen for lobular carcinoma. Researchers from BIG presented results of this comparison stratified between different subtypes of breast cancer. Although letrozole is superior to tamoxifen in all subgroups, the amount of benefit gained from this drug is dependent on the subgroup. Letrozole appears to provide a greater survival benefit for invasive lobular rather than invasive ductal carcinoma, as well as for both classic lobular and ductal tumours that are stratified into the luminal B subtype. There was a statistically significant reduction in events that threatened disease-free and overall survival with letrozole. These results suggest that letrozole should be preferred to tamoxifen, particularly for patients with luminal B tumours, but that further subgroup analysis is warranted and data from the sequential arm of the BIG 1–98 trial should be used to further explore the differences between luminal and luminal B tumours.

### Evaluating adjuvant tamoxifen at 15 years: Advantage for longer treatment

Researchers from the Adjuvant Tamoxifen: Longer Against Shorter (ATLAS) study of adjuvant tamoxifen therapy reported a clear advantage for the longer treatment. A study of over 7000 patients recruited from 36 countries showed that there is a small but significant benefit in both overall and progression-free survival (PFS) for patients who continue taking tamoxifen for ten years after surgery when compared with treatment for five years. The difference in mortality at 2%–3% was small for such a large trial but nevertheless it suggests that the longer regimen of adjuvant therapy with tamoxifen should become the standard. Little difference was observed between patients with different demographic and tumour characteristics, but in the longer term it would be useful to develop tools for the stratification and selection of patients by the degree of benefit that the longer treatment would offer.

### Prolonged survival with fulvestrant in patients with advanced breast cancer: Results from the CONFIRM trial

Angela di Leo from the Hospital of Prato, Italy, presented results from the large Phase III CONFIRM trial of the synthetic oestrogen receptor antagonist fulvestrant in postmenopausal women with advanced, hormone receptor positive breast cancer. Initial results indicate that the higher dose of 500 mg was associated with a median increase in overall survival of 4.1 months when compared with 250 mg. There was also a reduction of 19% in the risk of death and no increase in severe toxicity with the higher dose. Two to three per cent of patients are still receiving treatment with fulvestrant, and 90% have received one subsequent therapy. This survival increase was observed to be clinically relevant.

### Mutations in PIK3CA are linked to progesterone receptor expression in oestrogen receptor positive breast cancer

Researchers from the Tamoxifen Exemestane Adjuvant Multinational (TEAM) trial reported sequencing of the gene PIK3CA, which encodes the protein phosphatidylinositol 3-kinase, in oestrogen receptor positive breast tumours. This gene is most commonly found mutated in luminal A tumours, with mutations observed in approximately 40% of cases; in contrast, the RAS/RAF genes are uncommonly mutated in these tumours. Ten mutations of this gene were studied, and a correlation was observed between these mutations and increased expression of the progesterone receptor PgR. High numbers of mutations in this gene were associated with higher grade tumours. However, although mutations in phosphatidylinositol kinases in general have been associated with a high risk of tumour relapse, multivariate analysis did not reveal an association between mutations in PIK3CA and an increase in relapse risk.

### Promising results for a CDK inhibitor combined with letrozole

Cyclin dependent kinases are cell cycle regulators that have recently become prominent as targets for drugs against cancer. Promising results from preclinical and Phase I trials of a new selective inhibitor of this protein, PD 0332991, were presented at the SABC conference in 2009. Richard S. Finn from the University of California, Los Angeles has now supplemented these with “dramatic, significant and clinically meaningful” results of a Phase II study of this candidate drug in postmenopausal women with ER-positive/HER2-negative breast cancer, most with metastatic disease. All patients received letrozole in combination with either PD 0332991 or a placebo. The median PFS of all patients in the combination arm was 26.1 months, compared with 7.5 months for those receiving letrozole alone. Other criteria, including confirmed responses and “measurable clinical benefit” also showed a statistically significant benefit for the combination arm. The main side effect observed in patients receiving the study drug was neutropenia, but this could be controlled without need for growth factor treatment. These results indicate that a Phase III trial of PD 0332991 should go ahead, and one is planned to start later in 2013.

### Bevacizumab combined with endocrine therapy does not improve survival

Initial results were presented from the Phase III Letrozole/Fulvestrant and Avastin (LEA) trial of the angiogenesis inhibitor bevacizumab in combination with endocrine therapy as a first line therapy for postmenopausal women with hormone receptor positive breast cancer. In this trial, patients who had received no prior therapies were treated with an oestrogen receptor antagonist (letrozole or fulvestrant) alone or combined with bevacizumab. The primary end point was a median PFS of 18.4 months was recorded for patients receiving bevacizumab when compared with 13.8 months for those receiving hormone therapy alone. This difference was not statistically significant, and no significant difference was observed in the overall survival curve. This indicates that no significant benefit could be obtained by adding bevacizumab to endocrine treatment as a first-line therapy for postmenopausal women with hormone receptor positive disease.

### Prolactin humanised mice for luminal cancer xenografts

Haligeir Rui from Thomas Jefferson University in Philadelphia, Pennsylvania, United States, described a transgenic mouse model in which SCID mice are engineered to express human prolactin. This protein is a full antagonist of the mouse prolactin receptor. Xenografts of oestrogen receptor positive breast tumours from human patients grow faster in these mice than in other mouse models, and many of them metastatise to the mouse lungs. This mouse model is likely to prove useful in testing the response of this type of tumour to candidate drugs.

## GENERAL SESSION 2

### Sentinel lymph node surgery for node positive breast cancer

Up to 40% of breast cancer patients initially diagnosed as lymph node positive will convert to node negative after neoadjuvant chemotherapy. It will therefore, be useful to identify these patients in advance so they can be spared the more invasive axillary lymph node dissection. Judy Boughey from the Mayo Clinic in Minnesota, United States, presented results from the single arm Phase II American College of Surgeons Oncology Group (ACOSOG) trial of sentinel lymph node surgery in assessing node status. Approximately 700 patients with node positive breast cancer of all stages were enrolled in this trial; at least one sentinel lymph node could be identified in 639 patients and nodal status after neoadjuvant chemotherapy was identified correctly in 91.2% of these. It was found that the false negative rate could be lowered significantly if dual tracers were used and if a minimum of two sentinel lymph nodes were examined. Sentinel lymph node surgery can therefore be considered a useful tool for the detection of residual disease in lymph nodes following neoadjuvant chemotherapy for breast cancer.

### The SENTINA trial of sentinel node biopsy before or after neoadjuvant chemotherapy

The optimal timing of sentinel node biopsy in patients undergoing neoadjuvant treatment for breast cancer is unclear. The German SENTINA trial is a four arm prospective multicentre case-control study to compare the detection rates of sentinel node biopsy before and after neoadjuvant treatment and to determine false negative rates. A total of 1737 patients were enrolled and the overall detection rate was excellent. The false negative rate was significantly higher if node biopsy was carried out before rather than after neoadjuvant chemotherapy.

## Thursday, 6 December GENERAL SESSION 3

### Very young patients with breast cancer have better response to neoadjuvant chemotherapy

The response to neoadjuvant chemotherapy of breast cancer patients who are categorised as “very young” (up to and including 35 years of age) was analysed by selecting patients in this age group from the participants in eight trials of neoadjuvant therapy carried out by the German Breast Group. A total of 704 patients out of 8949 fell into this age category. The pathological complete response (pCR) rate was higher in patients under 35 than older ones (23.6% versus 15.7%; p < 0.001). The disease-free survival of young patients with all cancer types who achieved a pCR was never less than that of older ones. More young patients were diagnosed with triple-negative disease, and it is thought that this is the main driver of the good response to neoadjuvant chemotherapy in patients in this age group.

### Chemotherapy for patients with local and regional recurrences: Results of the CALOR trial

Stefan Aebi of Luzerner Kantonsspital in Luzern, Switzerland, presented results of the global chemotherapy as adjuvant for locally recurrent breast cancer (CALOR) trial testing whether adjuvant chemotherapy can help patients with an isolated local or regional recurrence of breast cancer. A total of 162 patients with local recurrences only were recruited onto this trial and randomised either to receive or not to receive adjuvant chemotherapy; all patients had surgery and radiation therapy. These patients had received a variety of prior treatments. The treatment regimen for those randomised onto the chemotherapy arm was determined, based on prior therapy. Five years after randomisation, disease-free survival rates were 69% for women in the chemotherapy arm, versus 57% for those who did not receive it (controls), resulting in a p-value of 0.045. Overall survival at 5 years was similar, at 88% for patients who received chemotherapy versus 76% for controls (p = 0.02). Adjuvant chemotherapy was most beneficial in patients diagnosed with triple-negative disease and least beneficial in oestrogen receptor positive patients, where the benefit failed to reach statistical significance. The researchers concluded that adjuvant chemotherapy should be recommended for breast cancer patients with completely resected local or regional recurrences, and particularly for those with oestrogen receptor negative disease.

### TACT 2 trial shows no benefit from dose dense chemotherapy

The TACT 2 trial in the UK compared standard and accelerated dose schedules of epirubicin in early breast cancer. A total of 4391 patients with this condition were randomised to receive four courses of epirubicin either every three weeks (standard treatment) or every two weeks (accelerated treatment), followed by pegfilgrastim, followed by standard chemotherapy. Other treatments could be given if suggested by the biology of the tumour. Approximately 60% of the patients had oestrogen receptor positive disease. After a median follow-up period of 49.3 months, 97.8% of the enrolled patients were still alive. There was no difference between the arms in the time to recurrence or in overall survival, and there was no apparent difference in treatment outcome between the subgroups. These results indicate that accelerated treatment with epirubicin offers no benefit over the standard regimen in early breast cancer.

### Ten-year follow-up shows that dose dense ETC confirms survival benefit in high risk breast cancer

Patients who were recruited into the AGO trial of dose dense chemotherapy for high risk breast cancer were followed up after ten years. This trial randomised patients with at least four positive lymph nodes to receive either dose dense or standard therapy with epirubicin, paclitaxel and cyclophosphamide (ETC). 79 per cent of the patients were oestrogen receptor positive. The intense dose schedule was found to give statistically significant superior overall survival after ten years in all patient subgroups.

### Risk of leukaemia after breast cancer diagnosis

The incidence of several types of leukaemia has been observed to rise in women who have previously been treated for breast cancer with chemotherapy. An analysis of 22,248 patient records from the National Comprehensive Cancer Network outcome database in the United States, showed that the risk of developing myelodysplastic syndrome and/or acute myelogenous leukaemia was raised following treatment with either chemotherapy plus radiotherapy or radiotherapy alone. The overall incidence of acute myeloid leukaemia in these patients was 0.5%, and stratification showed that the diseases were more frequent in young patients who had been treated with anthracyclines and alkylating agents.

### Genetic profiling of refractory triple-negative breast tumours

Justin Balko of Vanderbilt University, Nashville, Tennessee, United States, presented a deep sequencing and molecular profiling study of tumours from more than 100 patients with refractory, residual, triple-negative breast cancer. The sequences of 182 oncogenes and tumour suppressor genes were compared and approximately 90% of the tumours were found to bear mutations in genes that could possibly be targeted by various types of anticancer drugs, including kinase inhibitors and agents targeted to DNA repair pathways. Full data was available for 81 tumours, and the tumour suppressor P53 was found to be the most frequently altered gene followed by MCL1, which was frequently amplified, and the transcription factor MYC. Interactions between mutated MYC and MEK were found to predict poor survival.

## GENERAL SESSION 4

### Ten-year results of the START trial of hypofractionated radiotherapy

John Yarnold of the Royal Marsden Hospital, London, United Kingdom, presented results from the Standardisation of Breast Radiotherapy (START) trial after ten years of follow-up. Over 4500 patients with early breast cancer were randomised into two trials, each with two arms; one compared 25 fractions of post-surgery radiotherapy for five weeks with 15 fractions delivered over the same time period, and the other compared 25 fractions over five weeks with 15 fractions delivered over three weeks. The results of these trials, which were consistent with earlier reported results, showed no statistically significant difference in the relapse rate between the arms in either trial. Adverse effects on the tissue of the conserved breast were slightly lower in the regimens with the lower total radiation doses. These results indicate that the three-week, 15-fraction schedule for hypofractionated radiation, which has become the standard of care in the United Kingdom and is expanding into other countries, is just as effective as and marginally safer than regimens involving higher overall doses over longer time spans.

### Local recurrence in breast cancer: The TARGIT-A trial

Breast cancer most often recurs near the site of the primary tumour, indicating that radiotherapy targeted to that tumour site might be effective and have a reduced risk of cardiac and other side effects. Results were presented from an ongoing Phase III trial. Targeted Intra-operative Radiotherapy (TARGIT-A), which is comparing outcomes in breast cancer patients with a good prognosis who undergo either whole breast external beam radiotherapy (EBRT) or a single high dose of radiotherapy targeted to the site of the tumour. Since 2000, a total of 3451 women from 10 countries have been recruited into this trial. Early results suggest a trend towards reduced mortality in the targeted radiotherapy arm, although the size of the effect is small. The difference in mortality seems to be entirely due to second cancers and cardiac events, rather than to the original breast cancer; there was no difference between the arms in the small numbers of deaths due to breast cancer. No differences in outcome were observed when the patients were stratified by age, tumour size, grade, lymph node status or HER2 status.

### The EndoPredict score predicts metastases in oestrogen receptor positive, HER2-negative breast cancer

Breast tumours that are oestrogen receptor positive and herceptin receptor negative (ER^+^/HER2^– ^tumours) commonly give rise to late metastases. A group of researchers based in Vienna, Austria have developed a tool called EndoPredict to predict late recurrence in these tumours based on a score derived from the expression patterns of proliferative and ESR-1 related genes. A total of 1702 patients with breast cancer of this subtype were followed up for a median seven years and the likelihood of recurrence of each cancer was predicted using EndoPredict. This gene expression profile was found to be a good predictor of the risk of late relapse using either univariate or multivariate analysis, with increased sensitivity in the low-risk patients.

### Independent validation of the genomic grade index in predicting outcomes

The genomic grade index (GGI) is a 97-gene signature that has been developed to separate histological grade 2 breast tumours by predicted outcome. Results from a test of the validity of this index were reported in a subgroup of patients from the BIG 1-98 trial. Samples from a total of 883 patients yielded RNA of high enough quality for the GGI to be determined using PCR. Each sample was graded as either GGI1 (good prognosis), GGI3 (poor prognosis) or equivocal. These results were compared with the patients’ outcome and with levels of the antigen Ki67, high expression of which is also associated with poor prognosis. Both GGI and Ki67 levels were found to be significantly predictive of outcome, with 100% of patients graded GGI1, 94% of those graded intermediate and 85% of those graded GGI3 remaining free of distant metastases at the end of the study period.

### Ki67 expression levels predict response after neoadjuvant chemotherapy

Results presented from the GeparTrio trial of neoadjuvant chemotherapy have shown that expression levels of the antigen Ki67 can be both a prognostic and a predictive marker of response to neoadjuvant chemotherapy. Patients in this small single-centre study were stratified into three groups: low expression (<15% of cells showing expression); intermediate (15%–35% of cells) and high (>35%). High expression levels were found to be correlated with worse disease-free and overall survival, indicating that Ki67 is a negative prognostic marker and a positive predictive marker. Some differences were observed between tumour subtypes in the optimum cutoff levels used to define the different groups.

## Friday, 7 December GENERAL SESSION 5

### Biomarker analysis in the CLEOPATRA trial

Results from the Phase III Clinical Evaluation Of Pertuzumab And Trastuzumab (CLEOPATRA) trial of pertuzumab as a first line treatment for HER2-positive, metastatic breast cancer have already shown that adding this drug to a regimen including trastuzumab and docetaxel improves both progression-free and overall survival. The results presented at this meeting described the assessment of a panel of biomarkers in tumour and serum samples and the utility of this panel in predicting prognosis. Low levels of dHER2 in serum were associated with poor prognosis, while high levels of HER2 mRNA were associated with better outcomes. Mutations in the phosphatidylinositol 3-kinase gene PIK3CA were associated with worse prognosis than the wild-type form of this gene in both arms of the trial. PFS in the trastuzumab-only arm was 8.6 months for patients with mutated PIK3CA against 12.5 months those with wild-type PIK3CA, and the corresponding results for the dual-antibody arm were 13.8 and 21.8 months, respectively. The association between PIK3CA mutations and poor prognosis was particularly marked in patients with hormone receptor positive tumours. These results indicate that it would be useful to study trastuzumab in combination with inhibitors of this kinase.

### Prolonging adjuvant trastuzumab beyond one year yields no additional benefit in early stage, HER2+ breast cancer

The standard of care for patients with early stage, HER2 receptor positive breast cancer includes adjuvant therapy with trastuzumab for one year after surgery. The international, multicentre Phase III HERA trial enrolled 5102 patients with this type of breast cancer and randomised them to receive no adjuvant therapy, or trastuzumab for one or two years after surgery. The results presented at this meeting compared those patients who had received trastuzumab for one year with those who had received it for two. There was no difference in disease-free or overall survival between these two arms after four years of follow-up care. Primary cardiac adverse events were rare in both arms but marginally more common in the patients who received the adjuvant therapy for two years. Most of these, however, were transitory and reversible following the cessation of therapy. There was no statistically significant difference in outcome between patients with oestrogen receptor positive and negative disease. These results indicate that there is no additional benefit, and there may be some increased toxicity, in continuing adjuvant trastuzumab therapy for two years after surgery, and the study authors suggested that one year of this treatment should remain the standard of care.

### Length of treatment with adjuvant trastuzumab: The PHARE trial

The optimum length of adjuvant therapy with trastuzumab for early breast cancer has not yet been determined. Results were presented from the Phase III PHARE (Protocol for Herceptin^® ^as Adjuvant therapy with Reduced Exposure) trial that compared the standard treatment of 12 months of trastuzumab therapy with a shorter course of six months. 56% of the 3380 patients enrolled had oestrogen receptor positive disease; 73% received anthracyclines and taxanes as adjuvant therapy and 57% received taxanes concurrently with trastuzumab. Disease-free survival was 87 months in the 12-month therapy arm and 84 months in the six-month arm, which does not represent a statistically significant benefit for the longer course of treatment. Some differences were observed between patients with different oestrogen receptor status and chemotherapy modalities, but in general, the study showed that six months of adjuvant treatment with trastuzumab is not inferior to the standard 12 months of treatment in this patient group. The study is still ongoing and results from a longer follow-up period are awaited.

### High EGFR expression is associated with worse outcome in HER2-positive breast cancer

The epidermal growth factor receptor (EGFR) is known to dimerise with the herceptin receptor HER2, and its overexpression is thought to affect the effectiveness of trastuzumab therapy. This theory was tested by analysing the EGFR expression status of patients enrolled into the NCCTG (Alliance) N9831 trastuzumab trial. EGFR expression levels for the 3505 patients enrolled into this trial were obtained using quantitative immunofluorescence. The only difference in outcomes was observed in the arm in which patients received concurrent trastuzumab and chemotherapy; in this arm patients with high EGFR levels had a significantly worse prognosis. This data was confirmed using multivariate analysis. Therefore, these results confirm the hypothesis that high levels of EGFR decreases patients’ benefit from adjuvant treatment with concurrent trastuzumab.

### Benefit from trastuzumab in the adjuvant setting confirmed

An update of the joint analysis of the NSABP-31 and NCCTG 9831 trials of adjuvant trastuzumab was presented, giving survival results over a ten-year follow-up period. A total of 4036 patients were enrolled into these studies, of whom 50% were under 50 and about 45% had oestrogen receptor negative tumours. After a median follow-up of 8.4 years, the survival benefit obtained from trastuzumab had been maintained in all the subgroups. The risk of mortality was reduced by 37% and the risk of disease-free survival was reduced by 40% during this follow-up period in the patients who had received trastuzumab. Similar levels of benefit were seen in HER2-positive and negative disease.

### Genome sequencing identifies HER2 mutations as drug targets

Ron Bose from Washington University School of Medicine, St. Louis, Missouri, United States, presented results of gene sequencing of breast tumours with somatic mutations in the HER2 receptor but no overexpression of this gene. Bose and co-workers reviewed data from genome sequencing studies including almost 1500 patients and selected 25 tumours with this HER2 profile. This type of HER2 mutation was estimated to occur in about one per cent to two per cent of all breast tumours. Most of the mutations were located in the tyrosine kinase domain of the HER2 protein, with a smaller number in the domain involved in protein dimerisation. Tests in cell lines bearing these mutations showed that the tumour cells were more resistant to lapatinib than to neratinib, suggesting that neratinib might be a useful therapy for patients whose breast tumours bear these particular mutations in the HER2 gene. A Phase II trial of this drug in this patient subgroup is ongoing.

### Blocking the PI3K/AKT and EGFR/HER3 pathways in triple-negative breast cancer

About a third of breast tumours that are diagnosed as triple-negative overexpress the epidermal growth factor receptor (EGFR) and most of these tumours also show upregulation of the PI3K/AKT signalling pathway. Inhibition of the PI3K/AKT pathway with drugs often results in upregulation of the EGFR/HER3 pathway. It has been suggested, therefore, that dual targeting of these pathways with PI3K/AKT and EGFR inhibitors will prevent this upregulation. This hypothesis was tested by treating triple-negative breast cancer cell lines with these characteristics with inhibitors of PI3K and AKT and an antibody against EGFR and HER3. Combinations of drugs that target both pathways were found to be more effective than single drugs in reducing tumour volume and in downregulating the expression of phosphoAKT and PIK3Ca. These results suggest that it would be useful to test a similar combination of agents in the clinic with patients with triple-negative breast cancer whose tumours have these characteristics.

### Lapatinib resistance mediated by ER and bcl2 upregulation

Researchers from Houston, Texas, United States, presented the results of a Phase II trial of neoadjuvant lapatinib in patients with HER2-positive breast cancer. 49 patients with this type of breast cancer were treated with lapatinib followed by a combination of trastuzumab and docetaxel prior to surgery. Samples of the patients’ tumours were assessed for biomarker expression at several points during the treatment. An inverse correlation was observed between expression of HER2 and of the oestrogen receptor. Inhibition of HER2 by lapatinib resulted in increased expression of both the apoptosis regulator Bcl2 and oestrogen receptor. In order to overcome resistance to lapatinib in this tumours, it will be necessary to block both the oestrogen receptor and the Bcl2 pathways.

## GENERAL SESSION 6

### Isoform switching and mutation expression analysed by mRNA sequencing

Researchers from North Carolina reported results from sequencing the exomes, or the coding regions of the genomes, from a total of 728 breast tumour samples of all main subtypes, and from samples of normal tissue from the same patients. Genes in which statistically significant differences between isoforms expressed in the tumour and normal tissue samples were identified using hierarchical clustering. A significant level of isoform switching was observed that was independent of the subtype of breast cancer, and many of the isoforms found in the tumour tissue were associated with copy number changes.

### Cognitive problems may be present before chemotherapy

Magnetic resonance imaging during tasks involving memory was used to assess the cognitive function of breast cancer patients undergoing chemotherapy and radiotherapy and matched controls. The reported results showed that all patients showed less activity than the controls in the inferior frontal gyrus; a brain region that supports working memory. The patients also showed greater error rates in the test than the controls. Patients receiving radiotherapy performed better than those receiving chemotherapy, and their error rates decreased after treatment ended. Error rates and brain activation were also correlated with self-reported levels of fatigue, and patients in the chemotherapy group reported the highest levels of fatigue. The presenter suggested that as fatigue and the associated decline in cognitive function were present before the treatment of phenomenon of cognitive loss by chemotherapy that is referred to as “chemo brain” and it must be more complicated than is often thought. Women who experience cognitive problems during chemotherapy may benefit from prior interventions to treat cancer related fatigue and reduce psychological distress.

### Cognitive problems may be present before chemotherapy

Magnetic resonance imaging during tasks involving memory was used to assess the cognitive function of breast cancer patients undergoing chemotherapy and radiotherapy and matched controls. The reported results showed that all patients showed less activity than the controls in the inferior frontal gyrus; a brain region that supports working memory. The patients also showed greater error rates in the test than the controls. Patients receiving radiotherapy performed better than those receiving chemotherapy, and their error rates decreased after treatment ended. Error rates and brain activation were also correlated with self-reported levels of fatigue, and patients in the chemotherapy group reported the highest levels of fatigue. The presenter suggested that as fatigue and the associated decline in cognitive function were present before the treatment of phenomenon of cognitive loss by chemotherapy that is referred to as “chemo brain” and it must be more complicated than is often thought. Women who experience cognitive problems during chemotherapy may benefit from prior interventions to treat cancer related fatigue and reduce psychological distress.

### Results from the AZURE trial show high vitamin D levels associated with better prognosis

The Adjuvant Zoledronic Acid to Reduce Recurrence (AZURE) trial evaluated the effect of adding zoledronic acid to standard adjuvant therapy in stages 2 and 3 of breast cancer, and found that benefit from zoledronic acid was restricted to postmenopausal women. Serum from 827 participants in this trial was collected for biomarker analysis. The biomarkers analysed for prognostic and predictive value included 25-hydroxy vitamin D (25-OHD) and several markers of bone turnover. Levels of 25-OHD and of vitamin D were found to be associated with a better prognosis and particularly with better responses to zoledronic acid in postmenopausal women. Patients with low oestrogen levels were also shown to respond better to zoledronic acid, but neither of the bone turnover markers tested showed any prognostic or predictive value.

### The BEATRICE Trial of adjuvant bevacizumab for triple-negative breast cancer

Researchers involved in BEATRICE, a Phase III trial of adjuvant bevacizumab for triple-negative breast cancer, presented initial results from the trial. A total of 2600 patients with invasive but operable triple-negative disease and a median age of 35 were randomised either to receive chemotherapy plus bevacizumab followed by bevacizumab alone, or to receive standard chemotherapy only. Several different chemotherapy regimens were allowed. The primary endpoint was invasive disease-free survival. At the cutoff point, 82% of patients in the bevacizumab arm were alive without invasive disease, compared with 83% for patients in the control arm; this difference was not statistically significant. Furthermore, discontinuation of therapy and serious (grades 3–4) toxicity were more frequent in the bevacizumab arm than in the control arm. The most common adverse events were hypertension, proteinuria and cardiovascular events. Therefore, addition of bevacizumab to chemotherapy does not provide any statistically significant improvement in patients with triple-negative breast cancer. However, a large biomarker analysis is still underway to see whether any subgroups of patients who may benefit from this addition to their therapy can be identified.

### Eribulin offers no clear improvement over capecitabine in metastatic breast cancer

Results were reported from a large open label Phase III trial of the microtubule dynamics inhibitor eribulin against capectabine in patients with heavily pretreated, metastatic breast cancer. A total of 1102 patients who had received up to two prior treatments for metastatic disease were randomised to receive either eribulin or capecitabine alone. The primary end points were overall survival and PFS. Patients in the eribulin arm showed slight increases in both parameters; the medial overall survival was 15.9 months in the eribulin arm and 14.5 months in the capecitabine arm, and PFS was 4.1 months in patients receiving eribulin and 4.0 months in those receiving capecitabine. Neither of these differences was statistically significant. The reported toxicity of the two drugs was similar, although the types of adverse events differed. This trial has therefore failed to prove that eribulin is superior to capecitabine in metastatic breast cancer. However, a subgroup analysis seems to suggest that there may be a significant benefit in patients with the particularly aggressive triple-negative form of the disease. The median overall survival in patients with this form of cancer was 14.4 months with eribulin but only 9.4 months with capecitabine. Further analysis of erilubin in patients with triple negative and possibly other types of advanced breast cancer should go ahead.

### PDL1 predicts response to immune modulators

Researchers from the Neo-Sphere trial of the immune modulators trastuzumab and pertuzumab presented the results from an associated biomarker study. Tumour samples were taken from 387 of the patients with HER2-positive breast cancer who participated in this study before initiation of treatment and their gene expression profiles determined. The samples were taken equally from patients in all four arms of the study, which tested the effectiveness of pertuzumab and trastuzumab with and without docetaxel in the neoadjuvant setting. Genes known to be involved in immune responses were selected for further analysis. High expression levels of the gene PDL1, which encodes a protein that modulates apoptosis, were associated with higher levels of residual cancer, whereas pathological complete responses were more common in patients whose tumours had high expression levels of the PD-1 gene. This encodes a protein that forms complexes with PDL1 and that promotes cell differentiation. These results suggest that combining immune modulators with anti-HER2 antibodies might be a useful treatment option for patients with HER2-positive disease.

